# Investigation of the Spatiotemporal Responses of Nanoparticles in Tumor Tissues with a Small-Scale Mathematical Model

**DOI:** 10.1371/journal.pone.0059135

**Published:** 2013-04-02

**Authors:** Cheng-Ying Chou, Chih-Kang Huang, Kuo-Wei Lu, Tzyy-Leng Horng, Win-Li Lin

**Affiliations:** 1 Department of Bio-Industrial Mechatronics Engineering, National Taiwan University, Taipei, Taiwan; 2 Institute of Biomedical Engineering, National Taiwan University, Taipei, Taiwan; 3 Medical Engineering Research Division, National Health Research Institutes, Miaoli, Taiwan; 4 Department of Applied Mathematics, Feng Chia University, Taichung, Taiwan; University Hospital of Modena and Reggio Emilia, Italy

## Abstract

The transport and accumulation of anticancer nanodrugs in tumor tissues are affected by many factors including particle properties, vascular density and leakiness, and interstitial diffusivity. It is important to understand the effects of these factors on the detailed drug distribution in the entire tumor for an effective treatment. In this study, we developed a small-scale mathematical model to systematically study the spatiotemporal responses and accumulative exposures of macromolecular carriers in localized tumor tissues. We chose various dextrans as model carriers and studied the effects of vascular density, permeability, diffusivity, and half-life of dextrans on their spatiotemporal concentration responses and accumulative exposure distribution to tumor cells. The relevant biological parameters were obtained from experimental results previously reported by the Dreher group. The area under concentration-time response curve (AUC) quantified the extent of tissue exposure to a drug and therefore was considered more reliable in assessing the extent of the overall drug exposure than individual concentrations. The results showed that 1) a small macromolecule can penetrate deep into the tumor interstitium and produce a uniform but low spatial distribution of AUC; 2) large macromolecules produce high AUC in the perivascular region, but low AUC in the distal region away from vessels; 3) medium-sized macromolecules produce a relatively uniform and high AUC in the tumor interstitium between two vessels; 4) enhancement of permeability can elevate the level of AUC, but have little effect on its uniformity while enhancement of diffusivity is able to raise the level of AUC and improve its uniformity; 5) a longer half-life can produce a deeper penetration and a higher level of AUC distribution. The numerical results indicate that a long half-life carrier in plasma and a high interstitial diffusivity are the key factors to produce a high and relatively uniform spatial AUC distribution in the interstitium.

## Introduction

Delivery of chemotherapeutic nanodrugs to the targeted tumor cells from intravenous injection includes transport and distribution of nanodrug to tumors and other organs via system circulation, extravasation from tumor vasculature, and interstitial transport to reach individual tumor cells. The chemotherapeutic efficacy depends on the spatial and temporal concentration distribution of nanodrugs in the entire tumor, which is related to tumor micro-environments and physicochemical properties of nanodrug carriers. In addition, the toxicity of nanodrugs to normal tissues should also be taken into consideration.

The characteristics of tumor vasculature and interstitial space significantly influence drug delivery in solid tumors. In contrast to normal tissues, tumor vessels are leaky, chaotic, and non-homogeneously distributed [1]–[3]. Leaky vasculature and lack of a lymphatic system result in a higher vascular permeability and difficulty for nanodrug to clear. These lead to nanodrug accumulation in tumors more readily, i.e., the effect of “enhanced permeability and retention” [4], [5]. However, the spatial distribution of nanodrugs is not homogeneous in solid tumors, which mainly results from the decreased vascular density from the peripheral to the central region of tumor [6], [7]. Both the concentration of extracellular matrices and the size of intercellular space influence the transport of macromolecular nanodrugs in tumor tissues [8]. Conventional anticancer drugs are small molecules and are toxic to normal tissue due to poor tumor selectivity. Nanodrug carriers, such as liposome, micelle, dendrimer, etc., are developed to solve this problem [9]–[11]. In addition, the circulation time of nanodrug carriers is much longer than molecular drug [12], [13], and surface modification (e.g., polyethylene glycol) can further extend the circulation time of nanodrug carriers [12], [13]. On the other hand, the vascular permeability and interstitial diffusivity for nanodrug carriers are smaller than molecular drugs. A low vascular permeability and interstitial diffusivity will hinder the nanodrug transport in the interstitium of a tumor. Half-life in plasma, vascular permeability and interstitial diffusivity of nanodrug carriers are related to many physicochemical properties like size, charge, shape, etc. [12]–[14].

Modeling the effects of critical factors on the spatial and temporal responses of nanodrug carriers in tumor tissues can offer an insight into the efficiency of tumor chemotherapy. A mathematical model describing the delivery of monoclonal antibodies (mAb) in prevascular tumor nodule was constructed by Banerjee *et al.* [15], and the results of their study suggested that the mAb diffusivity and mAb binding site density in tumors played important roles in mAb delivery. Graff and Wittrup [16] analyzed the antibody targeting tumor spheroids by numerical methods, and the model provided a relationship between the molecular weight of antibodies and the area under interstitial drug concentration-time curve (AUC) of antibody in tumors. To be more elaborate, AUC is defined as the total area under the curve that describes the concentration of the drug in the interstitium as a function of time post injection. AUC is an important pharmacokinetic parameter that quantifies the extent of tissue exposure to a drug and the drug clearance from the body. AUC is considered to be more reliable in assessing the extent of the overall drug exposure than individual concentrations [17]. AUC plays an important role in toxicology [18], therapeutic efficacy [19]–[21], biopharmaceutics and pharmcokinetics [22] as it can be used to quantify the maximum tolerance exposure, to provide bioavailability information, and to determine pharmacokinetic parameters like clearance, etc.

In 2008, Goodman *et al.* [23] developed a mathematical model of nanoparticles penetrating into tumor spheroids, and their results suggested that particle size, particle binding, and porosity of target tissue were the crucial factors for nanoparticle delivery in tumors. Gasselhuber *et al.* [24] recently employed the compartment model to study the two-component liposomal drug, Doxorubicin (DOX). They used ordinary differential equations (ODEs) by assuming homogeneity of concentration in space for each compartment. Though more thorough compartments were considered, their model could not fully address the mechanisms of diffusion and convection, which are fundamentally important for the current model of drug transport in the tumor micro-environment. These previous models have pointed out some relevant factors regarding the nanodrug penetration in tumor spheroids. In practical tumor chemotherapy, the spatial and temporal concentration responses of nanodrugs in the entire tumor region, dependent on vascular density and transport parameters, are important indicators of the treatment efficacy and should be investigated in detail.

In this study, the vascular density of tumors and transport parameters, such as vascular permeability, interstitial diffusivity, and half-life time of macromolecular nanodrug carriers, were investigated by a mathematical model. Our model displays the spatial and temporal responses of macromolecular carriers with different transport parameters and vascular density in tumor tissues. The experimental results published by Dreher *et al.* were used to obtain the transport parameters of macromolecular carriers for the proposed mathematical model and we then extended these transport parameters to study their effects on the spatiotemporal distribution of macromolecular concentration in tumor tissues [14]. We also developed an auxiliary hydrodynamic model to compute the leaked flow rate and validated it with the case of normal tissues (Supporting Information S1). The parameters employed in the validation are contained in Table S1 in Supporting Information S1. This study provides a comprehensive insight into the optimal delivery of macromolecular nanodrug carriers in the entire tumors, and can facilitate the tailoring of nanodrug carriers.

## Methods

The primary goal of drug delivery is to increase the concentration and accumulative exposure of therapeutic agents in the tumor tissue. In other words, the larger the AUC in the tumor, the better. The delivery efficiency of a therapeutic agent is highly dependent on the internal structure of tumors. Different from normal tissue, the tumor structures are much more complicated and vary largely with respect to the size and type of tumors [25]. A highly irregular vascular network will make the already complex problem become intractable. Thus, the transport of therapeutic agents from the blood circulation to the tumor tissue is a rather complex process, and an insight into this mechanism can benefit the design and administration of treatments. To obtain such an insight, it is necessary to make some appropriate simplifications. Despite that the entire vascular structure is highly random, some spatial periodicity can be reasonably assumed at the end of this vascular hierarchy. Under this assumption, here we develop a mathematical model to investigate the joint effects of multiple factors mentioned above on the penetration of therapeutic agents within the tumor interstitium. The transport of various kinds of therapeutic agents is thoroughly studied here via numerical simulation to understand the inherent fundamental mechanism, which can definitely help researchers identify the critical factors relevant to the delivery of each drug carrier. The mathematical model and numerical method are detailed below followed by the computational result and its discussion.

### Geometric configuration

The heterogeneity of blood perfusion in tumors is caused by the uneven distribution of vasculature in neoplastic tissues [26]. The periphery of a tumor is usually well vascularized while semi-necrotic and necrotic regions are found with the increasing depth into the tumor. The interstitial pressure study of isolated tumors by Boucher *et al.* showed that the pressure rises rapidly in the periphery and soon reaches a maximum plateau value throughout the rest of a tumor. They also indicated that the maximum values were reached at a distance of 0.15 to 1.2 mm from the surface of most of the isolated tumors studied. Zero pressure gradient was maintained throughout the plateau within the tumor [27].

**Figure 1:**
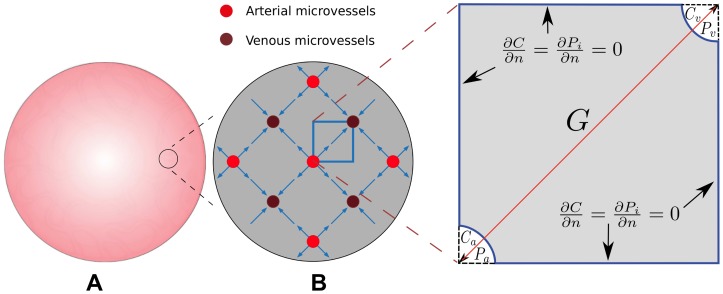
Illustration of a spherical tumor and schematic of the vascular distribution in a given tumor region. Illustration of a spherical tumor, and a darker region indicates a higher vascular density (subfigure A). A schematic of the vascular distribution in a given tumor region (subfigure B). The zoom-in region illustrates an equivalent block for simulating the transport of macromolecular carrier in tumor tissue. A smaller 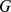 value indicates a larger vascular density.

In this work, we do not investigate the drug transport across the entire tumor, which is a very difficult problem both in modeling and simulation, but rather study the drug delivery behavior locally. Under the assumption that the microvessel network can be considered tightly arranged and periodically distributed like crystals, a small zoom-in region is considered here with negligible pressure gradient imposed from the surrounding tissue. In such a small region, the pressure gradient occurs only due to the source and sink of local, leaky microvessels. The two-dimensional cross-sectional view of the distribution of these microvessels is illustrated in Figure 1A with an arterial microvessel surrounded by four venous ones and *vice versa*. Applying symmetry, a square element of this periodic structure with a pair of arterial and venous microvessels located at two opposite corners of the square would be the geometric configuration for the current problem naturally, as shown in Figure 1B. The pressure distribution and the flow pattern in this small square area was investigated and they can be extended to a larger-scale region under symmetry and periodicity.

Here, as shown in Figure 1B, the vascular hydrostatic pressures inside the arterial and venous microvessels are denoted by 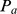 and 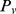, respectively, and the interstitial hydrostatic pressure is denoted by 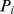. Similarly, the concentrations inside arterial and venous microvessels are denoted by 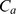 and 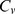, respectively. The distance between the centers of the two nearest arterial and venous microvessels is referred to as the vascular distance and denoted by 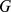 here. Generally speaking, 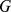 is determined by the vascular density with the vascular density being inversely proportional to 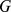. Usually vascular density is larger in the peripheral region of tumor, but smaller when deeper inside the tumor. Note that our small-scale model (a zoom-in local model) is general and can be applied to any region in a tumor characterized by different vascular density. A region near the tumor surface would have a higher vascular density, (i.e., small 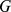 values), while a region away from the tumor surface would have a lower vascular density (i.e., large 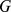 values) [28]. Though the pressure gradient is large in the periphery of tumor and small inside the tumor, this global pressure gradient can be neglected in the current local model, since 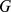 is typically from 100 to 400 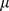
m only, and the pressure distribution in such a small square area is chiefly dominated by the difference between 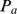 and 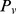.

### Mathematical models

#### Assumption within the microvessels

We assume that the injected drug is well circulated, and thus the concentrations along microvessels are set equal. The change of concentration is caused by tissue absorption, elimination through the lymphatic system and other physiological uptakes. The joint effects of these drug uptakes result in a temporal change of the drug concentration inside blood vessels, which can be approximated by fitting the experimental data. Consequently, the concentration decay, combining all the elimination effects in the body, can be described in terms of half-life in plasma of each drug carrier as,
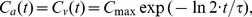
(1)where 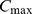 is the injection concentration of the drug and 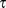 is the half-life time of the drug carrier in plasma.

In our work, the nanodrug delivery in tumor tissues was investigated. For convection, the drug is transported across the wall of arterial microvessel (high pressure area), travels through the tumor interstitium and is finally drained by venous microvessel (low pressure area). For diffusion, drug is diffused from high-concentration area (blood microvessels) to low-concentration one (interstitium). The whole mechanism of drug transport is the interplay between convection and diffusion with the wish that the AUC of drug is as large as possible in tumor interstitium. Convection would be the dominant transport mechanism for large molecules since they move slowly by diffusion. In contrast, small molecules diffuse faster and therefore their dominant transport mechanism is diffusion [25].

#### Governing equations in the transmural region

The leaky microvessels can be modeled as hollow cylinders with semi-permeable walls embedded in a porous medium (tumor interstitium). According to Starling's hypothesis, the net fluid flow across a vessel wall is given by
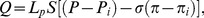
(2)where 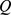 is volume flow of fluid across the vessel wall (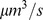); 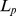 is the hydraulic conductivity (or the filtration coefficient) of the vessel (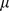
m/mmHg-s); 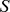 is the surface area of the vessel 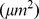; 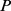 (mmHg) and 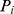 (mmHg) are the microvessel and interstitial fluid hydrostatic pressures (mmHg); 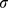 is reflection coefficient; and 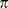 (mmHg) and 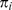 (mmHg) are the microvessel and interstitial osmotic pressures, respectively. The rate of solute transport across the blood vessel is described by Kedem-Katchalsky equation as
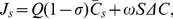
(3)where 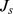 is the solute flux (mole/s); 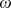 is microvascular permeability 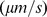, 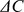 (mole/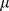
m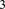) is the concentration difference across the vessel wall, and 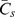 (mole/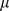
m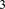) is the average concentration of solutions placed at both sides of the membrane as follows
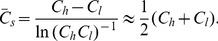
(4)where 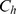 and 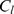 correspond to the high and low concentration values across the vascular wall, respectively. The first term of right-hand side of Equation (3) accounts for the convection, while the second term accounts for the diffusion.

#### Governing equations in the interstitial region

The flow in the interstitial area satisfies Darcy's law as [3]

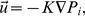
(5)where 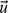 is the fluid velocity in the interstitium (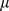
m/s), 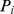 is the hydrostatic pressure for interstitium (mmHg), and 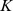 is the hydraulic conductivity (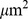/mmHg-s). Taking use of Darcy's law and the equation of continuity for incompressible fluid, the pressure in the interstitial region can be written as



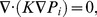
(6)with no-flux boundary condition (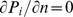, 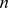 denoting the normal direction of the boundary) at all the vertical and horizontal boundaries due to symmetry and Dirichlet boundary conditions at arterial and venous microvessel walls as shown in Figure 1B. Consequently, Equation (6) was first utilized to solve for the pressure field, which was subsequently substituted into Equation (5) to determine the fluid velocity in the interstitium.

Provided the velocity distribution in the interstitium, determined from Equations (5) and (6), the concentration of drug carriers can be determined by solving the following transport equation
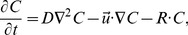
(7)where 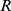 (1/s) is the additional absorption constant caused by tumor tissues; 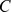 (mole/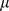
m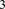) is the concentration of drug carrier in the interstitium, and 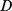 is the effective diffusion coefficient, which depends on the characteristic of the carrier (mainly the molecular weight) and the tumor structure. In the work hereafter, the magnitude of 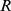 is also modeled relative to the size of the macromolecules. We assume that smaller dextran macromolecules will experience faster degradation in tumor tissues than the larger ones [29], [30]. Similar to the pressure field, the boundary conditions for 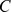 are no-flux boundary conditions, 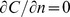, at vertical and horizontal boundaries in Figure 1B due to symmetry and are Robin boundary conditions at microvessel walls, which can be derived by equating the flux of solute concentration at the microvessel wall consisted of diffusion and convection with the transmural solute transport described in Equation (3) as



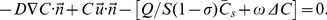
(8)Once velocity field is obtained through solving Equations (5) and (6), the drug carrier concentration in the tissues can be computed by Equation (7).

### Numerical method

**Figure 2:**
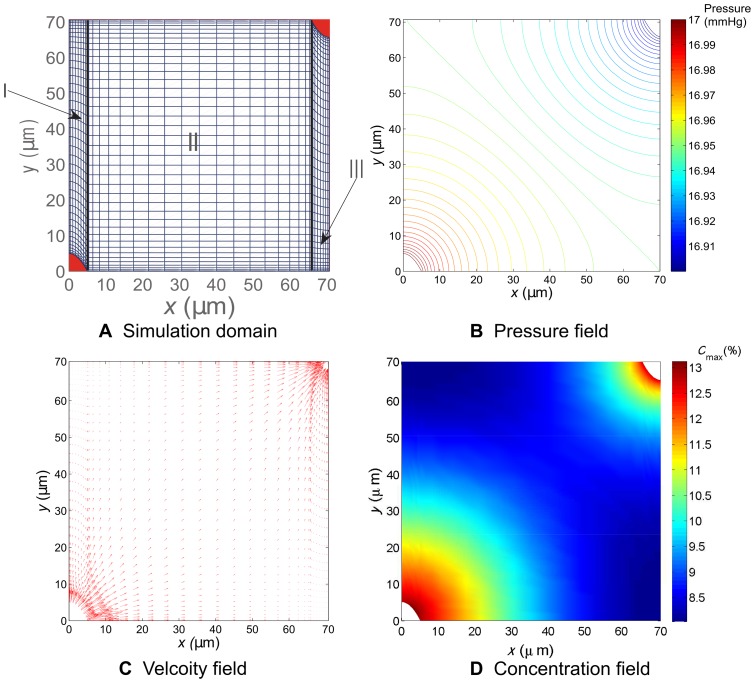
Illustration of the simulation domain and the computed pressure, velocity and concentration fields. Illustration of the simulation domain and the computed pressure, velocity and concentration fields. Subfigure A illustrates sub-domains I, II, and III, and their meshes for 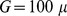
m. Subfigures B and C are the computed pressure and velocity fields, respectively. D. The concentration distribution for 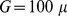
m at 100 min.

To compute Equation (6), under the geometric configuration shown in Figure 1, we embedded Equation (6) into a time-dependent problem as described in Equation (S1) in Supporting Information S1. The computer simulation was carried out by employing the method of lines (MOL) and multi-block Chebyshev pseudospectral method to compute Equation (S1) to reach its steady state. Once the velocity field is obtained, we can compute Equations (7) and (8) similarly. The details of our numerical method are elaborated in Supporting Information S1. Interested readers can refer to this supplement for more information. Here we demonstrate our numerical method by computing an example of pressure, velocity, and drug concentration distributions shown in Figure 2, in which we can see, in the presence of the pressure gradient, the transport of the drug carrier is driven by both the flow convection and diffusion.

## Results and Discussion

It is important to have an anticancer nanodrug access all tumor cells in lethal quantities to avoid tumor recurrence caused by certain cells that remain alive after treatments. Therefore, the penetration depth and accumulative exposure of an anticancer nanodrug in the interstitial region of tumors are crucial to the tumor's overall drug exposure, in particular for distant cancer cells residing away from the vasculature. This means we wish the spatial distribution of AUC to be of a high level and as uniform as possible at the same time. In general, the nanodrug distribution within a tumor is determined by its supply, the vascular permeability, interstitial transport of the nanodrug, and nanodrug-cell interactions. Below, we investigate and discuss how AUC distribution depends on vascular density, vascular permeability, interstitial diffusivity, and half-life in plasma. Though the computational domain shown in Figure 1B is two-dimensional, for the discussions hereafter we only consider one-dimensional AUC spatial distribution with the one-dimensional coordinate along the center-to-center line connecting arterial and venous microvessels.

### Obtaining vascular permeability and interstitial diffusivity of dextran macromolecules in tumor tissues from experimental data

**Figure 3:**
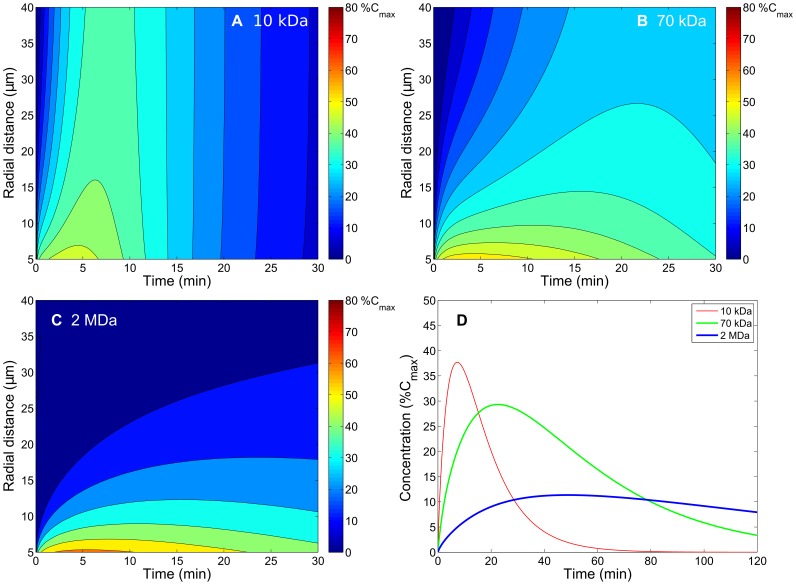
Fitting results of spatiotemporal and spatially averaged concentration responses of macromolecular carriers (dextran). Fitting results of spatiotemporal and spatially averaged concentration responses of macromolecular carriers (dextran) corresponding to molecular weights (A. 10 kDa, B. 70 kDa and C. 2 MDa) in the tumor interstitium with a single blood vessel by tuning the vascular permeability and interstitial diffusivity for each carrier to match [14]. Subfigure D contains a plot through the average concentration for each carrier.

To determine the physiological parameters of macromolecular dextrans in tumor tissues, we used the current model to fit the experimental results of spatial-temporal distributions for different molecular weights of dextrans [14]. The parameters, including vascular permeability, interstitial diffusivity and half-life in plasma, were tuned to yield the spatial-temporal responses of concentration distributions that resemble those observed in Figure 6 of [14]. Figure 3A–3C shows the spatiotemporal responses of concentration distributions corresponding to 10-kDa, 70-kDa, and 2-MDa dextrans transported into tumor interstitium respectively from a single microvessel with the radial distance denoting the distance from the center of the microvessel. The responses of spatially averaged extravascular concentrations were also computed and shown in Figure 0D, where the red, green, and blue curves denote the concentrations for 10-kDa, 70-kDa, and 2-MDa dextrans, respectively. After carefully tuning the simulation parameters, the final fitting values for 3.3-kDa, 10-kDa, 70-kDa, and 2-MDa dextrans are listed in Table 1, and the values of 16-MDa dextran were extrapolated from them. Table 1 generally shows that vascular permeability and interstitial diffusivity decrease as molecular weight of dextran increases, but half-life in plasma increases as molecular weight of dextran increases.

**Table 1:** The characteristics for dextrans of different molecular weights.

Vascular permeability	Interstitial diffusivity	Plasma half-life time
(*μ*m/s)	(*μ*m^2^/s)	(min)
*ω*_3.3k_ = 1.890	*D*_3.3k_ = 30.83	*τ*_3.3k_ = 7.35
*ω*_10k_ = 1.280	*D*_10k_ = 17.28	*τ*_10k_ = 8.17
*ω*_70k_ = 0.392	*D*_70k_ = 3.01	*τ*_70k = _23.77
*ω*_2M_ = 0.085	*D*_2M_ = 0.26	*τ*_2M = _35.14
*ω*_16M_ = 0.060	*D*_16M_ = 0.16	*τ*_16M = _37.25

The values for 3.3-kDa, 10-kDa, 70-kDa, and 2-MDa dextrans were fitted from Figure 6 of [14] while those for 16-MDa dextran were extrapolated from the former four.

### Effect of vascular density on the AUC distribution of dextran macromolecules in tumor tissues

**Figure 4:**
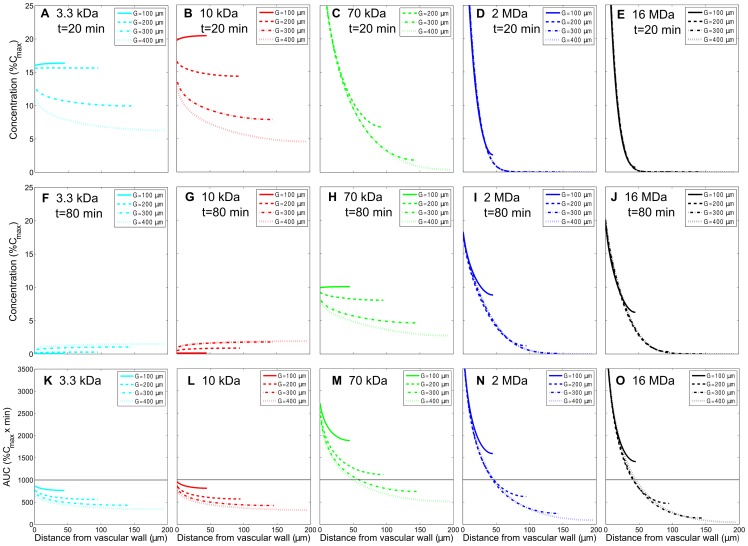
Effect of vascular density on the spatial distribution of instantaneous concentration and the accumulative exposure. The effect of vascular density on the spatial distribution of instantaneous concentration response of various macromolecular carriers (MW: 3.3 kDa, 10 kDa, 70 kDa, 2 MDa, 16MDa) at 20 min and 80 min and their accumulative exposure (in the radial distance, up to 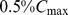) in the tumor interstitium. A–E and F–J are concentration distributions at 20 min and 80 min, respectively. K–O correspond to the accumulative exposures. The horizontal lines show the value of AUC equal to 1000 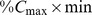. The permeability, interstitial diffusivity, and plasma half-life time for dextrans of different molecular weights in this case are listed in Table 1. (Note that the solid curve corresponding to 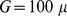
m is not shown in subfigure C because its values are beyond the range of those plotted).

**Figure 5:**
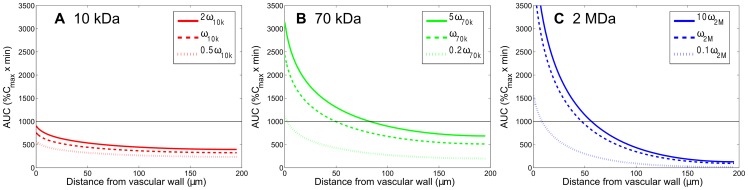
Effect of vascular permeability of carriers on the spatial distribution of accumulative exposure. The effect of vascular permeability of carriers on the spatial distribution of accumulative exposure (in the radial distance, up to 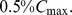) (MW: 10 kDa, 70 kDa, 2 MDa) in the tumor interstitium. The horizontal lines show the value of AUC equal to 1000 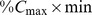. The diffusion coefficient and plasma half-life time in this case are listed in Table 1 corresponding to each molecular weight.

**Figure 6:**
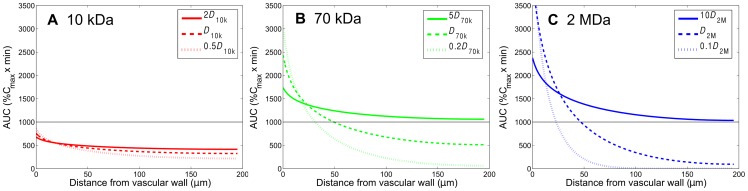
Effect of interstitial diffusivity of carriers on the spatial distribution of accumulative exposure. The effect of interstitial diffusivity of carriers on the spatial distribution of accumulative exposure (in the radial distance, up to 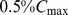) (MW: 10 kDa, 70 kDa, 2 MDa) in the tumor interstitium. The horizontal lines show the value of AUC equal to 1000 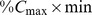.

**Figure 7:**
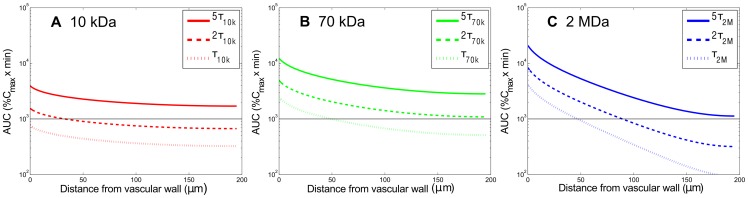
Effect of the plasma half-life time of carriers on the spatial distribution of accumulative exposure. The effect of the plasma half-life time of carriers on the spatial distribution of accumulative exposure (up to 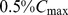). The horizontal lines show the value of AUC equal to 1000 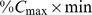. The permeability and interstitial diffusivity in this case are listed in Table 1 corresponding to each molecular weight.

The physiological parameters of macromolecular dextrans shown in Table 1 were employed to investigate the effect of vascular density, characterized by vascular distance 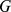, on the spatial AUC distribution of dextran macromolecules in tumor tissues. AUC reflects the actual tumor tissue exposure to the anti-tumor drug after its administration. Here, four vascular distances (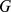 values) of 100, 200, 300, and 400 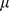
m, corresponding to tumor tissues with high to low vascular densities, were considered. In order to elucidate the progression of dextran accumulation, the spatial distributions of instantaneous concentrations of dextrans with 3.3 kDa, 10 kDa, 70 kDa, 2 MDa, and 16 MDa at 20 and 80 min, after drug is injected to microvessels, are shown in Figure 4A–4E and in Figure 4F–4J, respectively, for different vascular densities. The associated spatial AUC distributions for the entire duration of accumulative exposure are shown in Figure 4K–4O. Note that the horizontal lines in Figures 4–7 serve as the reference value to show the relative distribution of dextrans and can be chosen to correspond to an effective threshold value of the drug accumulation. In this study, the entire time course of accumulative exposure is defined as the time at which the extravascular concentration decreases to less than 0.5% 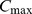, with 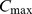 denoting the initial dextran concentration in plasma. As shown in Figure 4K–4O, 3.3-kDa and 10-kDa dextrans have more uniform distributions but with lower levels of AUC compared with 70-kDa, 2-MDa, and 16-MDa dextrans due to their larger interstitial diffusivity but smaller half-life in plasma. 70-kDa dextran has a more uniformly distributed AUC than 2-MDa and 16-MDa dextrans do, while they all have about the same level of AUC averages in space. By comparing Figure 4K–4O, it is apparent and comprehensible that AUCs of all dextrans with various molecular weights are less uniform in the lower vascular density case, particularly for larger macromolecules. If we consider the AUC average in space, the middle-sized dextran (70-kDa) has the largest value. It indicates that middle-sized drug carrier performs best in drug exposure in tumor interstitium under the interplay of permeability, diffusion, convection and plasma decay characterized by half-life in plasma.

Furthermore, Figure 4K–4O also shows that there is no significant difference of AUC distributions between 3.3-kDa and 10-kDa dextrans, as well as between 2-MDa and 16-MDa dextrans. Hence, we will only consider 10-kDa, 70-kDa and 2-MDa dextrans with a low vascular density (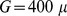
m) for investigating the effects of vascular permeability, interstitial diffusivity and half-life in plasma on AUC distribution later. In this study, we focused on the investigation of localized drug distribution and its accumulative exposure related to vascular density, molecular weight and other parameters, and hence we used a small-scale transport model with uniformly distributed vasculature and showed the concentration and AUC distributions between two closest vessels. However, the vascular density is not uniformly distributed in a three-dimensional tumor and macromolecules always diffuse from a higher concentration region to a lower one. This indicates that the actual AUC in a tumor region with high vascular density is supposedly a little bit lower than those shown in Figure 4K–4O; on the other hand, the AUC in a low vascular density region should be a bit higher than the simulated result. The AUC distributions of various macromolecules in Figure 4K–4O were grouped with vascular densities from 100, 200, 300, to 400 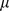
m, and hence the AUCs for other vascular densities are perceptible in these figures and able to obtain with a suitable fitting.

### Effect of vascular permeability on the AUC distribution of dextran macromolecules in tumor tissues

To study the influence of vascular permeability of dextran on their AUC distributions, we simulated the conditions with the vascular permeability both increased and decreased by different multiples of their respective values in Table 1 for 10-kDa, 70-kDa, and 2-MDa dextrans while keeping other parameters the same. The AUC distributions, calculated down to 0.5% 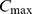 in the interstitial region between two microvessels with a vascular distance of 400 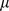
m are shown in Figure 5. Subfigure A displays the AUC for 10-kDa dextran with 2-, 1-, and 1/2-fold of permeability values while the permeability variations for 70-kDa (Figure 5B) and 2-MDa (Figure 5C) dextrans are from 1/5- fold to 5-fold and from 1/10-fold to 10-fold, respectively. We can see that larger vascular permeability, i.e. more leaky microvessels, results in a higher AUC than those with lower vascular permeability. The enhancement or reduction of vascular permeability has a particularly significant impact on the extravasation of larger particles like 2-MDa dextran and this is reflected on the uniformity of their AUCs. The average values of AUC in Figure 5 and their coefficients of variation (CVs) are shown in Table 2. Denoted as the ratio of the standard deviation to average [19], CV is related to the uniformity of AUC distribution in space. The smaller the CV value is, the more uniform the distribution. CV values in Table 2 indicate that larger particles or smaller permeability will result in a less uniform AUC distribution, and this is consistent with the findings shown in Figure 5.

**Table 2:** The average AUC and coefficient of variation (CV) values corresponding to different permeability of carriers shown in Figure 5.

Dextran MW	AUC*_kω_* ; *CV_kω_*	AUC*_ω_* ; *CV_ω_*	AUC*_ω/k_* ; *CV_ω/k_*
10 kDa	439.14; 13.5%	365.67; 13.7%	272.98; 13.8%
70 kDa	865.85; 31.5%	660.40; 31.7%	302.32; 32.7%
2 MDa	481.43; 96.4%	398.20; 98.4%	123.52; 122.9%

CV is defined as the ratio of the standard deviation (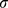) to the averaged value (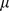): CV = 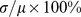. The 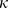 values for 10-kDa, 70-kDa, and 2-MDa dextrans are 2, 5, and 10, respectively.

The vascular permeability depends on the properties of particles (size, charge and configuration, etc.) and the vessel wall (pore size and density, charge and arrangement, etc.). As the particle size increases, the permeability decreases and becomes zero when the particle size is larger than the pore cut-off size. Nanoparticles that are larger than albumin are most likely to transport through intercellular junctions since inter-endothelial junctions in tumors can be as large as hundreds of nanometers to a few micrometers [7], [32]. Hydrophilic solutes and macromolecules use intercellular junctions and intracellular fenestrations, and macromolecules may also use vesicular transport. Tumor blood vessels have larger pores, and the vascular permeability and hydraulic conductivity are significantly higher than normal tissues [5], [33], [34]. Hence nanoparticles extravasate in the tumor tissue mainly from these large pores. Additionally, cationic nanoparticles preferentially target tumor vessels and display higher permeability compared with their anionic or neutral counterparts [35], [36]. It is also worth noting that not all tumor vessels are leaky for nanoparticles, and this is due to a heterogeneous distribution of pore sizes, which results in heterogeneous extravasation and delivery [37]. In this situation, the nanoparticle distribution will be similar to a localized region with a lower vascular density. Furthermore, the vascular permeability in tumors depends on the tumor location and varies with time in response to treatment [38].

### Effect of interstitial diffusivity on the AUC distribution of dextran macromolecules in tumor tissues

To investigate the influence of interstitial diffusivity of dextran macromolecules on their AUC distributions, we simulated the conditions with various interstitial diffusivities while the other parameters remain the same. The spatial distributions of AUC (calculated down to 0.5%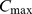) in the interstitial region between two microvessels with a vascular distance of 400 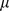
m are shown in Figure 6 for 10-kDa, 70-kDa, and 2-MDa dextrans, and the solid, dashed, and dotted curves denote enhanced, original, and reduced diffusivities, respectively. The enhanced diffusivity for 10-kDa, 70-kDa and 2-MDa dextrans are 34.56 (2×), 15.05 (5×) and 2.6 (10×) 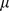
m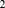/s, respectively. On the other hand, the reduced values of diffusivity for 10-kDa, 70-kDa and 2-MDa dextrans are 8.64 (0.5×), 0.62 (0.2×) and 0.026 (0.1×) 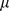
m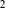/s, respectively. From Figure 6, we can see that AUC is more uniform and at the same time with higher level when diffusivity is enhanced, as anticipated. Table 3 gives the average and CV values of these AUC distributions. CV values indicate that enhancement of diffusivity promotes the nanoparticles delivery in the tumor interstitium, thereby resulting in a more uniform AUC distribution (i.e., smaller CV).

**Table 3:** The average AUC and CV values corresponding to different interstitial diffusivity of carriers shown in Figure 6.

Dextran MW	AUC*_kD_* ; *CV_kD_*	AUC*_D_* ; *CV_D_*	AUC*_D/k_* ; *CV_D/k_*
10 kDa	445.05; 6.9%	365.67; 13.7%	270.35; 26.5%
70 kDa	1138.13; 6.8%	660.40; 31.7%	225.39; 123.3%
2 MDa	1264.69; 11.7%	398.20; 98.4%	39.32; 613.9%

The 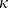 values for 10-kDa, 70-kDa, and 2-MDa dextrans are 2, 5, and 10, respectively.

The transport of nanoparticles through the interstitial matrix depends on diffusion and convection [18]. Convection depends on the gradient of interstitial fluid pressure and it is found negligible compared with diffusion due to small pressure difference at interstitial edges adjacent to blood vessels. Of course, this will not be true at the periphery of tumor where the pressure gradient is large, but this situation is beyond the scope of the current local model. Therefore, the main particle transport mechanism within tumor interstitium is diffusion. The movement of diffusing nanoparticles depends on the particle's properties (size, charge, configuration, etc.) and the physiochemical properties of the interstitial matrix [6]. Charged particles that develop electrostatic attraction or repulsion with charged components of the interstitium will hinder the particle's diffusion. The work of Dellian *et al.* indicated that the microvascular permeability to the positively charged molecules is higher than the permeability to the negative ones [36]. After being extravasated, however, the charged particles diffuse more slowly than neutral particles [39]. Therefore, we can anticipate that positive charges will result in enhanced microvascular permeability, but charges in general reduce diffusion in the interstitium.

### Effect of half-life in plasma on the AUC distribution of dextran macromolecules in tumor tissues

To examine the influence of half-life of dextran macromolecules in plasma on the AUC distribution, we simulated with half-lives increased by 5-fold and 2-fold of their original values for 10-kDa, 70-kDa, and 2-MDa dextrans, keeping other parameters unchanged. Figure 7 shows that the level but not uniformity of AUC distribution is significantly increased for all dextran macromolecules, which was no surprising since more dextrans leaked to the interstitium when they decayed more slowly in the plasma. The spatial averages of these AUC are provided in Table 4 and again it shows that the enhancement of AUC generally coincides with the enhancement of half-life. On the other hand, the spatial distribution of AUC, indicated by CV value, is hardly affected by the change of half-life. This is due to the fact that the enhancement of plasma half-life only prolongs the circulation time of drug carriers, but does not expedite their transportation in tissues.

**Table 4:** The average AUC and CV values corresponding to drug carriers of different half-lives shown in Figure 7.

Dextran MW	AUC*_5τ_* ; *CV_5τ_*	AUC_2*τ*_ ; *CV_2τ_*	AUC*_τ_* ; *CV_τ_*	AUC*_5τ_/AUC_τ_*	AUC*_2τ_* ; *AUC_τ_*
10 kDa	1843.04; 13.7%	734.93; 13.7%	365.67; 13.7%	5.04	2.00
70 kDa	3346.59; 31.2%	1333.19; 31.4%	660.40; 31.7%	5.06	2.02
2 MDa	2188.085; 88.7%	845.69; 92.1%	398.19; 98.4%	5.49	2.12

**Figure 8:**
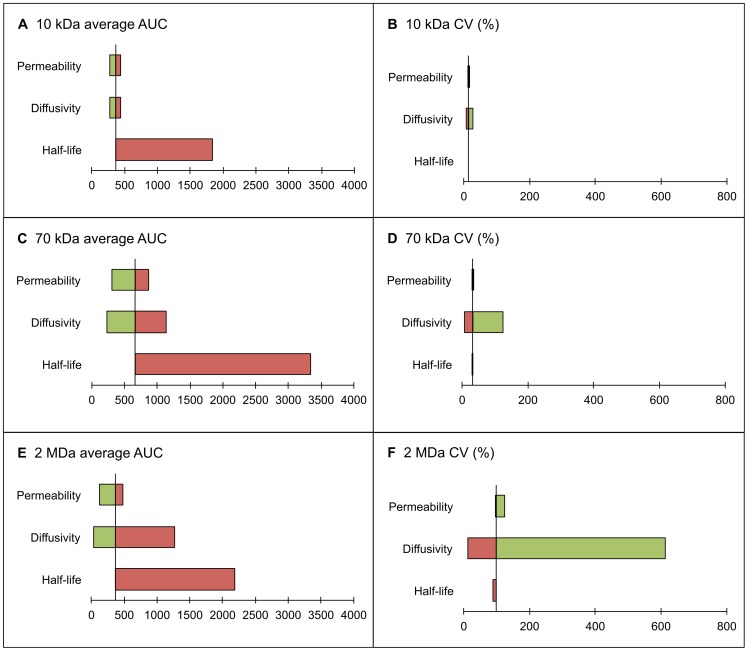
Tornado diagrams displaying the impact of carrier characteristics on the spatial distribution of accumulative exposure. The tornado diagrams of the impact of vascular permeability, interstitial diffusivity and plasma half-life time of carriers on the spatial distribution of accumulative exposure (up to 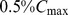). The average AUC and CV of the AUC distribution for 10-kDa dextran are presented in subfigures A and B. The corresponding diagrams for 70-kDa and 2-MDa dextrans are contained in subfigures C–D and E–F, respectively. The high and low values of the parameters correspond to the highest and lowest values contained in Tables 2, 3, 4, and are illustrated in red and green bars, respectively.

It is observed from Figure 8 that the plasma half-life and interstitial diffusivity are the key factors that govern the AUC value and its uniformity. The observed trend is general and applies to any nanodrug. The plasma half-life can be measured readily; however, the interstitial diffusivity is difficult to determine. The common practice is to measure the nanodrug's diffusivity in water and estimate its interstitial diffusivity based on its shape, charge, collagen content in tumor tissue, etc. The presence of binding sites reflects the condition similar to when the interstitial diffusivity is reduced. It does not significantly change the total accumulation of anti-cancer drug in the tumor region, but re-shapes its distribution. We can anticipate a higher AUC value in the perivascular region and lower one in the distal regions away from vessels.

To summarize, here we took dextrans as model macromolecules for nanodrug carriers and used different molecular weights of dextrans to study their spatiotemporal concentration distribution in tumor tissues after injection, focusing on the resulting spatial AUC distribution, which represents the accumulative exposure of tumor tissues to nanodrugs. We numerically analyzed the transport of nanodrugs by a simplified geometric model of tumor vasculature, which proved versatile for characterizing different conditions and different regions of tumors according to changed parameters.

In particular, we studied how vascular density, vascular permeability, interstitial diffusivity, and half-life in plasma affect the spatiotemporal concentration response and the associated AUC for dextran macromolecules in different molecular weights. The results indicate: 1) A higher vascular density means a shorter transport distance between vessels for sources of dextran macromolecules, and hence a higher and relatively more uniform spatial AUC distribution can be produced. Also the effect of vascular density on the spatial AUC distribution is more pronounced for large dextran macromolecules than small ones. 2) A medium-sized dextran macromolecule has the best performance among all three kinds of dextrans with various molecular weights considering both the level and uniformity of spatial AUC distribution in tumor interstitium. 3) A large dextran macromolecule possesses a long half-life while it results in a shallow penetration with a very high AUC in the perivascular region due to its low diffusivity, and the condition gets much worse for a tumor region with lower vascular density. 4) A small dextran macromolecule has the most uniform distribution of AUC among all due to its high interstitial diffusivity, but has the lowest level of AUC distribution due to short half-life in plasma. 5) Enhancement of vascular permeability implying more leaky vessels helps elevate the level of AUC for all three kinds of dextrans, but has little effect on the uniformity of distribution of AUC. 6) Enhancement of interstitial diffusivity would elevate the level of AUC and at the same time make AUC more uniform for all three kinds of dextrans. 7) A longer half-life of dextran macromolecules in plasma can produce a deeper penetration and a much higher AUC distribution.

A small dextran macromolecule generally has much better uniformity of spatial distribution of AUC due to large interstitial diffusivity compared to larger macromolecules. However, the level of its AUC is rather low because of its short half-life in plasma. Appropriate surface modification of dextran macromolecules may be able to extend the dextran macromolecule half-life in blood. Alternatively, we may physically or chemically transform large nanoparticles, when they enter tumor interstitium, to small ones that have greater vascular permeability and interstitial diffusivity to enable transport deeper into tumor tissues [40], [41]. This helps to yield a high AUC distribution in distal regions away from vessels while taking advantage of the long half-life of large nanoparticles inside the plasma to obtain a high level of AUC distribution. Ways to achieve this include using hyperthermia to increase nanoparticle permeability and diffusivity [42], using focused ultrasound with microbubbles to disrupt the vascular walls to increase nanoparticle permeability [43], [44], using collagen proteins to improve interstitial diffusivity of dextran macromolecules, and modifying the architecture of water-soluble polymer to improve half-life and diffusivity [45], [46]. A recent study by Peiris *et al.* developed a nanoparticle-based drug modified by multicomponent nano chains, which help anticancer drug delivery into deep interstitial and avascular regions [47]. Their results confirmed the claims of this work and indicated the key factors for a profound and homogeneous delivery of drug throughout the majority of a tumor are 1) a long circulation time, 2) permeation through the leaky tumor vasculature, i.e. high vascular permeability, and 3) drug release deep into cancer cells, which can be achieved by nanoparticles with high diffusivity.

## Conclusion

We present a small-scale mathematical model to study the concentration and AUC distributions for localized tumor regions under various conditions. The results clearly depict 1) the limitation of nanoparticle delivery in the local tumor tissues, 2) the treatable domain for a nanoparticle when the tumor and particle properties are given, and 3) the potential improvement when vascular permeability, interstitial diffusivity and half-life in plasma are enhanced. The current model is a two-dimensional model and can only be used to analyze the delivery and transport of nanoparticle-based drugs in a local tumor region with uniformly distributed blood vessels, which is well justified by its small scale. With the assorted results from various vascular densities in Figure 4, one may be able to perceive the concentration and AUC distributions in a three-dimensional tumor with heterogeneous vascular properties through the current small-scale model.

The effectiveness of cancer tumor treatment depends on the delivery of therapeutic agents to all tumor cells in different regions of a tumor in order to help avoid tumor regrowth and development of resistant cells. This study numerically elucidates the barriers to drug transport in the tumor tissues to assess methods that aim to achieve a higher and more uniform AUC distribution in the tumor tissues within regions of different vasculature. Thus we provide a better understanding of significant factors that contribute to therapeutic strategies aiming to improve passive and/or active tumor chemotherapy.

## Supporting Information

Supporting Information S1 Table S1, The physiological parameters of the normal tissue used to validate our numerical method.
(PDF)
