# Supplementary material for: Investigation of the Spatiotemporal Responses of Nanoparticles in Tumor Tissues with a Small-Scale Mathematical Model
Source: PLoS One. 2013 Apr 2;8(4):e59135. doi: 10.1371/journal.pone.0059135 (PMC3615073; doi:10.1371/journal.pone.0059135)
Supplement: Supporting Information S1 — Table S1, The physiological parameters of the normal tissue used to validate our numerical method. (PDF) [file pone.0059135.s001.pdf]

Supporting Information

Numerical methods

To compute Equation (6), under the geometric configuration shown in Figure 1, in a conventional finite-difference or finite-element way requests a structured or unstructured body-fitted mesh generation at first. This pre-processing is not a trivial job and usually takes a serious amount of man and computer hours to do so especially when a good-quality mesh is demanded. After discretization, Equation (6) would end up with a large sparse linear system, which can be solved directly by some sparse solvers. However, this approach would request huge computer memory. An alternative is to solve the linear system by iteration methods, which do not demand large computer memory, but sometimes suffer poor convergence due to ill-conditioning. The convergence usually can be improved by pre-conditioning, but it takes skills to choose a good pre-conditioner and the associated iteration method. To avoid all the difficulties above, here we embed Equation (6) into a time-dependent problem as follows,

$$\frac{\partial P_i}{\partial t} = \nabla \cdot (K \nabla P_i), \tag{S1}$$

and compute Equation (S1) until the steady-state is reached. Method of lines (MOL) is employed to compute Equation (S1). The basic idea of MOL is first to semi-discretize a time-dependent problem in space. This will end up with a set of coupled ordinary differential equations (ODE), which can be efficiently solved by many well-developed ODE solvers.

Here we applied the highly accurate multi-block Chebyshev pseudospectral method to discretize Equation (S1) in space. We first divided the physical domain shown in Figure 1 into three parts, denoted by sub-domains I, II, and III, as shown in Figure 2A. Since Chebyshev pseudospectral method can only be implemented on a square domain $[-1,1] \times [-1,1]$, we need to employ coordinate transformation to each physical sub-domain, denoted by $(x_j, y_j), j \in \{\text{I, II, III}\}$, to rectangular computational domains, denoted by (ξ_j, η_j), with $-1 \leq \xi_j, \eta_j \leq 1, j \in \{\text{I, II, III}\}$, respectively. Sigma transformation, frequently used in hydraulics, is applied to transform sub-domains I and III to rectangular ones.

Chebyshev pseudospectral method with Gauss-Lobatto grids (CPGL) has been a popular high-order numerical method for spatial discretization of partial differential equations [1]. To illustrate the simple idea of CPGL together with MOL, here we will use one-dimensional heat conduction problem as an example

$$\frac{\partial w}{\partial t} = \alpha \frac{\partial^2 w}{\partial x^2}, \quad t \in (0, \infty), \quad x \in (-1, 1). \tag{S2}$$

Note that when Equation (7) or Equation (S1) is considered, w will correspond to C or P_i. In basic theory of CPGL, if $w_N(x)$ is the projection of $w(x)$ into a polynomial space with degree less than or equal to N through Chebyshev polynomials as basis functions, then $w(x)$ can be well approximated by $w_N(x)$ when N is large. Actually, $w_N(x)$ converges exponentially to $w(x)$ as $N \to \infty$, provided $w(x)$ is smooth enough. $w_N(x)$ can be expressed through interpolation as

$$w_N(x) = \sum_{k=0}^{N} w(x_k) L_{N,k}(x), \tag{S3}$$

where $L_{N,k}(x)$ is the k-th Lagrange interpolation polynomial of degree N,

$$L_{N,k}(x) = \frac{(x - x_0)(x - x_1)\cdots(x - x_{k-1})(x - x_{k+1})\cdots(x - x_N)}{(x_k - x_0)(x_k - x_1)\cdots(x_k - x_{k-1})(x_k - x_{k+1})\cdots(x_k - x_N)}, \tag{S4}$$

with the interpolation node x_k being Chebyshev Gauss-Lobatto collocation points,

$$x_k = \cos\left(\frac{k\pi}{N}\right), \quad k = 0, 1, \cdots, N. \tag{S5}$$

Similarly, $w'_N(x)$ and $w''_N(x)$ will be good approximations of $w'(x)$ and $w''(x)$, respectively, and can be obtained through differentiation of Equation (S3),

$$w'_N(x) = \sum_{k=0}^{N} w(x_k) L'_{N,k}(x), \tag{S6a}$$

and

$$w''_N(x) = \sum_{k=0}^{N} w(x_k) L''_{N,k}(x). \tag{S6b}$$

where $'$ and $''$ denote the first and second derivatives. In the spirit of collocation method, we are only interested in function values on those collocation points. Therefore,

$$w'_N(x_l) = \sum_{k=0}^{N} w(x_k) L'_{N,k}(x_l), \quad l = 0, 1, \cdots, N \tag{S7a}$$

and

$$w''_N(x_l) = \sum_{k=0}^{N} w(x_k) L''_{N,k}(x_l), \quad l = 0, 1, \cdots, N, \tag{S7b}$$

where $L'_{N,k}(x_l)$ and $L''_{N,k}(x_l)$ are so-called 1st and 2nd order collocation derivative matrices usually denoted as $D^{(1)}$ and $D^{(2)}$. The entries of $D^{(1)}$ and $D^{(2)}$ can be found in Ref. [2]. In fact, $D^{(2)}$ can be well approximated by $D^{(1)}D^{(1)}$.

Equations (7) and (S1) will further turn into a set of differential-algebraic equations (DAE) with the algebraic equation part from boundary conditions which are independent of time. This DAE system can be solved by many well-developed DAE solvers, and here we employed ode15s, a popular MATLAB script, to solve this DAE system [3]. Besides boundary conditions mentioned above, extra interface conditions between sub-domains rising from domain decomposition are also required. They are simply continuity of P_i, C, and their normal derivatives. The current MOL+CPGL approach did show a great efficiency in computing Equations (7) and (S1), since it does not request huge number of grids to gain enough accuracy. Also Gauss-Lobatto collocation mesh tends to cluster near boundary and interface, as shown in Figure 2A. This may also help to resolve possible boundary layer economically.

Auxiliary hydrodynamic model

In order to validate our numerical method, we further developed the hydrodynamic model in the interstitial region. From Starling law, the pressure drops across arterial and venous walls are proportional to the transmural volume flow rate as

$$\frac{Q_a}{L_p S} = \left[(P_a - P'_a) - \sigma (\pi_a - \pi_i) \right], \tag{S8}$$

and

$$\frac{Q_v}{L_p S} = \left[(P'_v - P_v) - \sigma (\pi_i - \pi_v) \right], \tag{S9}$$

where subscripts "a" and "v" are employed to indicate the capillaries at arterial and venous ends, respectively. The capillary osmotic pressures are assumed to be $\pi_a = \pi_v \simeq iCRT$, with i and C denoting the van't Hoff factor and drug carrier concentration in capillaries, respectively. While the capillary pressure P_a or P_v can be measured in practice, the interstitial hydrostatic pressures P'_a or P'_v in the tumor interstitium right adjacent to vessel walls of arterial and venous microvessels respectively are yet to be determined.

To determine the values of $P_a' - P_v'$, we carried out intensive simulations to obtain the interstitial flow rate (Q_{tissue}) as an empirical function of the interstitial pressure drop ($P_a' - P_v'$), hydraulic conductivity (K), capillary radius (r), and vascular distance (G), which was found to be

$$\frac{Q_{\text{tissue}}}{2.021 \times K \times r^{2.745} G^{-1.42}} = (P_a' - P_v'), \tag{S10}$$

with $\mathrm{R}^2 = 0.9639$. Given the vascular distribution in our simulation model, the volume flow rates across the arterial capillary wall, interstitial space and venous capillary wall are equal to each other, i.e.,

$$Q_a = Q_v = Q_{\text{tissue}} = Q. \tag{S11}$$

By summing Equations (S8)-(S10), it yields the pressure difference between arterial and venous capillaries yields

$$P_a - P_v = Q\left[\frac{2}{L_p S} + \frac{1}{2.021 \times K \times r^{2.745} G^{-1.42}}\right]. \tag{S12}$$

Alternatively, the transmural and interstitial flow rate is described by

$$Q = \frac{P_a - P_v}{\frac{1}{2.021 \times K \times r^{2.745} G^{-1.42}} + \frac{2}{L_p S}}. \tag{S13}$$

Once Q is determined by Equation (S13), P_a' and P_v' can then be determined from Equations (S8) and (S9) respectively, which concludes the Dirichlet boundary conditions setup for P_i when solving Equation (S6) or Equation (S1) alternatively. From knowledge of normal tissue parameters listed in Table S1, we validated Equation (S13) by the computed volumetric flow rate $Q = 8.08 \times 10^{-7} \mu\mathrm{m}^3/\mathrm{s}$, which is comparable to the value of $9.8 \times 10^{-7}\ \mu\mathrm{m}^3/\mathrm{s}$ reported in Ref. [4]. This demonstrates the validity of our mathematical model and numerical method. Consequently, our method can help to identify the contributions of some key factors in the drug delivery and may facilitate the design of therapy strategies for tumors.

References

1. Canuto C, Hussaini M, Quarteroni A, Zang T (1988) Spectral methods in fluid dynamics. Springer-Verlag.

2. Peyret R (2002) Spectral methods for incompressible viscous flow. Springer Verlag.

3. Trefethen L (2000) Spectral methods in MATLAB. Society for Industrial Mathematics.

4. Fournier R (1999) Basic transport phenomena in biomedical engineering. Hemisphere Pub.

Table

Table S1. The physiological parameters of the normal tissue used to validate our numerical method.

Property	Value
Arterial and venous capillary radii (r_a, r_v)	5 μm
Capillary pressure at arterial end (P_a)	30 mmHg
Vascular osmotic pressures (π)	28 mmHg
Interstitial fluid pressures (P_i)	-3 mmHg
Interstitial osmotic pressures (π_i)	8 mmHg
Capillary pressure at venous end (P_v)	10 mmHg
